# Chromogenic *in situ *hybridization to detect EGFR gene copy number in cell blocks from fine-needle aspirates of non small cell lung carcinomas and lung metastases from colo-rectal cancer

**DOI:** 10.1186/1756-9966-29-125

**Published:** 2010-09-15

**Authors:** Giovanni Simone, Anita Mangia, Andrea Malfettone, Vincenza Rubini, Michele Siciliano, Anna Di Benedetto, Irene Terrenato, Flavia Novelli, Marcella Mottolese

**Affiliations:** 1 Pathology Department, "Giovanni Paolo II" National Cancer Institute, via Hahnemann 10, Bari, Italy; 2 Clinical Experimental Oncology Laboratory, Giovanni Paolo II National Cancer Institute, via Hahnemann 10, Bari, Italy; 3 Pathology Department, Regina Elena National Cancer Institute, via Elio Chianesi 53, Rome, Italy; 4 Epidemiology Department, Regina Elena National Cancer Institute, via Elio Chianesi 53, Rome, Italy; 5 UT Radiation Biology and Biomedicine, ENEA C.R. Casaccia, Via Anguillarese 301, Rome, Italy

## Abstract

**Background:**

Several studies demonstrated that epidermal growth factor receptor (EGFR) gene copy number (GCN) correlates to the response to tyrosine kinase inhibitors in non small cell lung cancer (NSCLC) and to anti-EGFR monoclonal antibodies (MoAbs) in metastatic colorectal cancer (CRC). In the presence of lung nodules, cytology is often the only possible diagnostic approach. Chromogenic *in situ *hybridization (CISH) is an alternative technique to fluorescence *in situ *hybridization (FISH), but its feasibility in detecting EGFR GCN in cell blocks from fine-needle aspiration cytology (FNAC) of lung nodules has not yet been established.

**Methods:**

We evaluated the feasibility of CISH on 33 FNAC from 20 primary NSCLC (5 squamous carcinomas, 8 large cell carcinomas and 7 adenocarcinomas) and 13 lung metastases from CRC.

**Results:**

Of the 33 FNAC analyzed by CISH, 27 (82%) presented a balanced increase in EGFR gene and chromosome 7 number: 10 cases (30%) showed a low polysomy, 15 (45%) a high polysomy and 2 (6%) NSCLC were amplified. No significant differences between NSCLC and CRC lung metastases were found in relation to disomic or polysomic status. In addition, no correlation between EGFR GCN and EGFR immunohistochemical overexpression was found. Furthermore, we compared CISH results with those obtained by FISH on the same samples and we found 97% overall agreement between the two assays (k = 0.78, p < 0.0001). Two cases were amplified with both assays, whereas 1 case of NSCLC was amplified by FISH only. CISH sensitivity was 67%, the specificity and positive predictive value (PPV) was 100%, and the negative predictive value (NPV) was 97%.

**Conclusions:**

Our study shows that CISH is a valid method to detect EGFR GCN in cell blocks from FNAC of primary NSCLC or metastatic CRC to the lung.

## Introduction

Epidermal growth factor receptor (EGFR) is a member of the erbB family of tyrosine kinases (TK) receptor proteins, that play an important role in tumor progression [1]. In fact, the binding EGFR/ligand leads to activation of the TK, thus inducing cell growth, inhibition of apoptosis, angiogenesis, invasion and metastasis [2]. EGFR overexpression in non small cell lung cancer (NSCLC) and colorectal cancer (CRC) is a frequent event related to a poor outcome [3]. In the last few years, many clinical trials have proven the efficacy of EGFR-targeted therapies in the management of several cancers, including breast, colon, pancreas, head and neck, renal, and lung carcinomas. Multiple therapeutic strategies have been developed to target EGFR, including monoclonal antibodies (MoAbs), tyrosine kinase inhibitors (TKI), ligand-toxin conjugates, and antisense oligonucleotides. Cetuximab and panitumumab are two MoAbs which are active against the ligand binding site of EGFR with high specificity and higher affinity for EGFR than the natural ligands TGF-α and EGF, and are now considered as one standard option for patients with advanced CRC in the first or second line of treatment [4,5]. Indeed, the anti-EGFR erlotinib and gefitinib have undergone extensive clinical testing demonstrating clinical activity in NSCLC [6].

In this context, there is a need for methods enabling response prediction in order to select those patients most likely to benefit from treatment. Therefore, the diagnostic approach of pathologists is changing, leading to an integrated morphological and molecular diagnosis.

EGFR overexpression does not seem a good predictor of response to treatment both in NSCLC and CRC [7,8], even though some controversial results are reported [9]. According to poor clinical information obtained from the immunohistochemistry (IHC), the interest in EGFR gene status increased after Moroni et al [10] proposed that in CRC the response to anti EGFR treatment with cetuximab is related to EGFR gene copy number (GCN) and Lynch et al [11] showed that, in advanced NSCLC, in-frame deletion or missense mutations in the EGFR TK domain can predict the response to therapy with gefinitib.

In addition, several authors [12,13] reported that, in metastatic CRC (mCRC), an increased EGFR GCN or mutations of genes (i.e. k-ras) responsible for downstream signalling are important determinants of response or resistance to anti-EGFR antibodies, such as cetuximab and panitumumab. Specifically, cetuximab has proven efficacy in the treatment of mCRC, but also in NSCLC with squamous cell histology [14].

Although fluorescence *in situ *hybridization (FISH) is the "gold standard" method to detect EGFR gene amplification, this technique presents some disadvantages since the fluorescent signal is not stable and morphological features are difficult to visualize. In contrast, chromogenic *in situ *hybridization (CISH) utilizes a peroxidase reaction to detect the locus of interest and can be interpreted by standard light microscopy in the context of morphology [15].

In the majority of lung neoplastic nodules, cytology is often the only possible diagnostic approach. Nevertheless, the cytological diagnosis of pulmonary nodules sampled by fine-needle aspiration cytology (FNAC) presented three main problems for the pathologist: a) the small amount of cellular specimens, b) the correct characterization of tumor histotype, and c) the report of biological information predictive of targeted therapy response. Conventional cytology can often provide insufficient material to answer these problems, while the availability of cell blocks allowed to perform multiple analyses as IHC, CISH/FISH and eventually gene mutations [16].

In a retrospective series of 33 pulmonary tumors, we investigated the feasibility and reliability of CISH performed in cell blocks obtained from FNAC, to detect EGFR gene copy number both in primary NSCLC and mCRC lung nodules. In addition, we compared CISH to FISH and IHC results.

## Materials and methods

### Patients and samples

Cell blocks from paraffin embedded FNAC of 33 lung neoplastic nodules were retrospectively selected from the Pathology Department Archives of the National Cancer Institute of Bari, Italy.

Twenty primary lung carcinomas, 18 from male and 2 from female patients, and 13 metastatic lung nodules from CRC (10 males and 3 females) were included in this study. Five of the 20 NSCLC were squamous cell carcinomas (SCC), 8 large cell carcinomas (LCC), and 7 adenocarcinomas (ADC). The median age of patients was 67 (range: 31-84 years).

FNAC samples were obtained with a CIBA 22-gauge needle (length 15 cm), and the aspiration procedure was performed under computed tomography (CT) guidance. All patients provided their written consent for use of the samples for research purposes.

### Cell Block Procedure

Cell blocks were prepared spinning the FNAC cellular specimens, fixed in 10% buffered formalin, at 1000 revolutions per minute for 10 minutes. After centrifugation, the sediment was re-suspended in 95° ethyl alcohol for 10 minutes and submitted to a second centrifugation. Then, the packed sediment was removed with a spatula and wrapped in lens paper. The wrapped sediment was embedded in paraffin according to conventional histological techniques after a short processing cycle with xylene.

Five consecutive 3-4 μm thick sections were cut from cell block of all 33 cases and processed by IHC to evaluate EGFR expression and by CISH and FISH to analyze gene amplification. The cytological slides were reviewed by a pathologist (GS), who verified the diagnosis and the percentage of neoplastic cells.

### Immunohistochemistry

The immunohistochemical assay for EGFR expression was performed on tissue sections from cell blocks using the EGFR PharmDx kit (Dako, Milan, Italy).

The deparaffinized and rehydrated sections were pre-treated in an enzyme solution (Proteinase-k) at room temperature (RT) for 5 minutes. After the block of endogenous peroxidase activity, the sections were incubated with EGFR MoAb (IgG1, clone 2-18C9, Dako) for 30 minutes, employing 3'3-diaminobenzidine (DAB) as a chromogenic substrate. Sections were slightly counterstained with Mayer's hematoxylin and mounted in aqueous mounting medium (Glicergel, Dako).

Dako control slides were used as positive controls and the negative control was performed by omitting the application of the primary antibody.

IHC scoring was based on the membrane immunoreactivity, according to the American Joint Committee [17]: 0, no reactivity, 1+, weak reactivity, 2+, moderate reactivity, 3+, strong reactivity.

### Chromogenic in situ hybridization

Formalin fixed paraffin embedded (FFPE) sections were deparaffinized, dehydrated, air dried, and heated in boiling tissue heat pre-treatment buffer for 15 minutes using a SPoT-Light^® ^FFPE reagent kit (Zymed, Histoline, Milan, Italy). Enzymatic digestion was performed using SPoT-Light^® ^FFPE digestion enzyme (Zymed) for 2-3 minutes at RT. After dehydration, histological slides were air dried and the ready-to-use double-stranded DNA digoxygenin-labelled EGFR probe (Zymed) or the biotin labelled chromosome 7 centromeric probe (Zymed) were applied. Denaturation was performed by incubating the slides, covered with a CISH cover-slip, on a 96°C heating block for 5 minutes, and hybridization was performed by placing the slides in a humidity chamber at 37°C overnight. After removing the cover-slips, a stringent wash was performed in 0.5× saline-sodium citrate buffer at 80°C for 5 minutes. The endogenous peroxidase activity and unspecific staining were blocked by applying 3% hydrogen peroxide and the CAS-Block™, respectively. A mouse antidigoxygenin antibody was added to the slides hybridized with EGFR probe for 45 minutes at RT followed by incubation with a polymerized peroxidase-goat anti-mouse antibody (Dako) for 45 minutes at RT. On the FFPE tissue slides, the colorimetric signal of chromosome 7 centromeric probe was improved by incubating the slides with a mouse antibiotin antibody (Dako) for 45 minutes at 37°C. A DAB chromogen substrate system was used to generate a sensitive signal that could be viewed with a Nikon ECLIPSE 55i transmission light-brightfield microscope (Nikon, Amstelveen, The Netherlands) after Mayer's haematoxylin counterstaining.

### Fluorescence in situ hybridization

FISH was performed using the LSI EGFR (SpectrumOrange™), a locus-specific probe for the EGFR human gene locus (7q12) and the chromosome enumeration probe (CEP 7, SpectrumGreen™) for alpha-satellite DNA located at the centromere (7q11.1-q11.1) (Vysis, Inc., Downers Grove, IL). The assay was carried out according to the manufacturer's instructions. Shortly after deparaffinization, the FFPE specimens were incubated in the pre-treatment solution (82°C, 30 minutes) and then digested with protease (37°C, 15 minutes). After washing, the slides were counterstained with 4',6-diamidino-2 phenylindole (DAPI) and analyzed using a fluorescent microscope. An average of 30 nuclei was counted for each case. The EGFR gene copy number, chromosome 7 copy number, and the average EGFR gene to chromosome 7 signal ratio were reported as FISH genetic variables.

### CISH and FISH analysis

The CISH and FISH results were assessed using the categories proposed by Daniele et al. [18]. Four majors patterns were identified: balanced disomy (1.6-2.0 gene and chromosome 7 in all cells), balanced trisomy (2.2-3.0 gene and chromosome 7), balanced polysomy (3.1-4.4 gene and chromosome 7), low amplification (gene-to-chromosome 7 ratio 2.1-3.0), and high amplification (gene-to-chromosome 7 ratio > 3.0). We considered the presence of at least a group of 10 neoplastic cells showing gene gain as the positive cut off.

The CISH and FISH signals were read by 2 investigators (MM and ADB) independently from the results of the other assays.

### Statistical Analysis

Agreements between the test results (IHC, CISH and FISH) were estimated using the Cohen's k test and its relative 95% confidence interval (95% CI). Specificity, sensitivity, negative and positive predicted value (NPV and PPV, respectively), concordance and the 95% CI of the CISH assay were estimated considering the FISH result as the gold standard. Significance was assessed at 5% level. The statistical software package used for this analysis was SPSS for Windows (version 17.0; SPSS Inc., Chicago IL, USA).

## Results

### EGFR gene copy number according to tumor histotype

**Figure 1:**
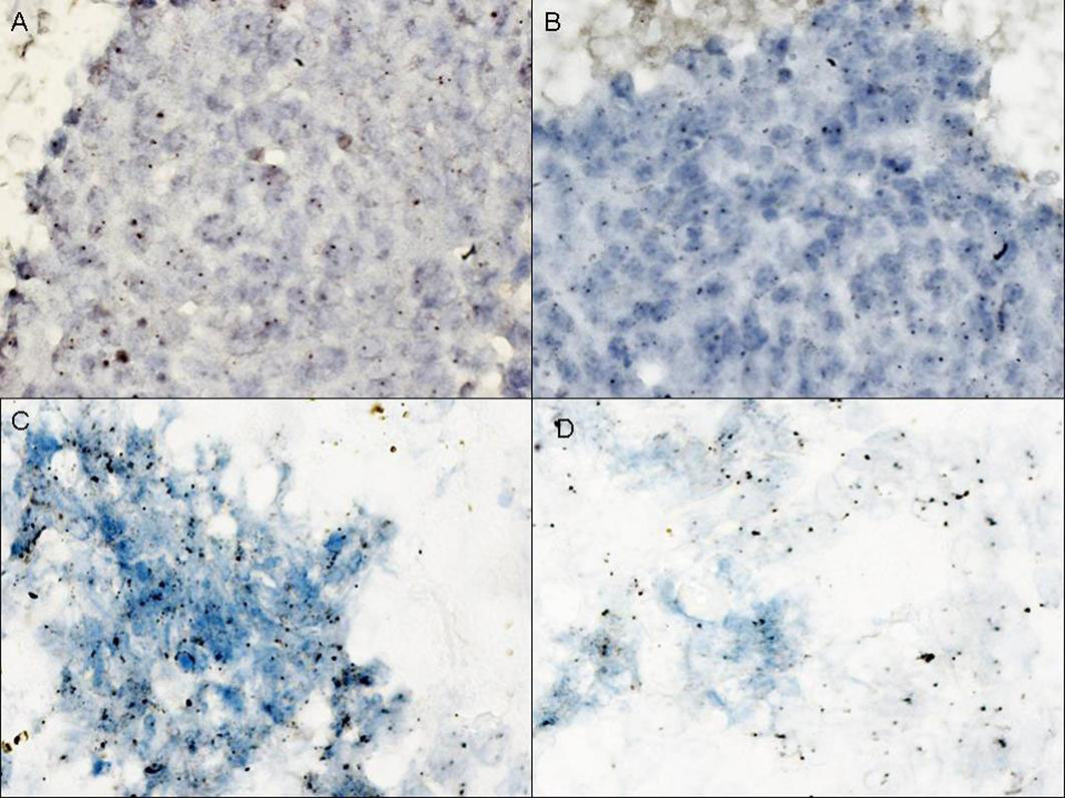
**EGFR CISH analysis on non small cell lung carcinoma**. Two different patterns of gene and chromosome 7 copy number obtained by CISH on cell blocks prepared from two different Lung Carcinoma FNAC: (A) EGFR not amplified and (B) paired chromosome 7 disomy; (C) EGFR gene amplification with a clustered pattern and (D) trisomy of chromosome 7. Original magnification ×1000.

The CISH analysis was performed successfully on cell blocks of 20 NSCLC and 13 pulmonary mCRC. Of the 33 FNAC samples analyzed, 27 (82%) presented an increased EGFR GCN. In detail, as summarized in Table 1, 6 cases (18%) were disomic (1.6-2.0 balanced gene and chromosome 7) (fig 1A, B), 10 (30%) presented low polysomy (trisomy: 2.2-3.0 balanced gene and chromosome 7) and 15 (45%) high polysomy (3.1-4.4 balanced gene and chromosome 7). The 2 amplified NCSLC (gene-to-chromosome 7 ratio ≥ 2), were 1 ADC and 1 LCC (fig 1C, D). No significant differences between NSCLC and pulmonary metastases from CRC, were observed in relation to the disomic or polysomic status.

**Table 1:** Distribution of EGFR gene copy number evaluated by CISH according to tumor histotype

Histotype	N° of cases	Disomy	Trisomy	Polysomy	Amplified
ADC	7	1	2	3	1
LCC	8	2	1	4	1
SCC	5	1	3	1	0
mCRC	13	2	4	7	0

Total	33	6	10	15	2

ADC: adenocarcinoma; LCC: large cell carcinoma; SCC: squamous cell carcinoma; mCRC: metastatic colo-rectal cancer; Disomy: 1.6-2.0 balanced gene and chromosome 7; Trisomy: balanced 2.2-3.0 gene and chromosome 7; Polysomy: 3.1-4.4 balanced gene and chromosome 7; Amplified: gene-to-chromosome 7 ratio ≥ 2

### Immunohistochemistry results and comparison between CISH and FISH

Positive immunoreactivity for EGFR was observed in 72% of the 33 cases and was distributed as follows: 65% NCSLC and 84% lung metastasis from CRC.

**Figure 2:**
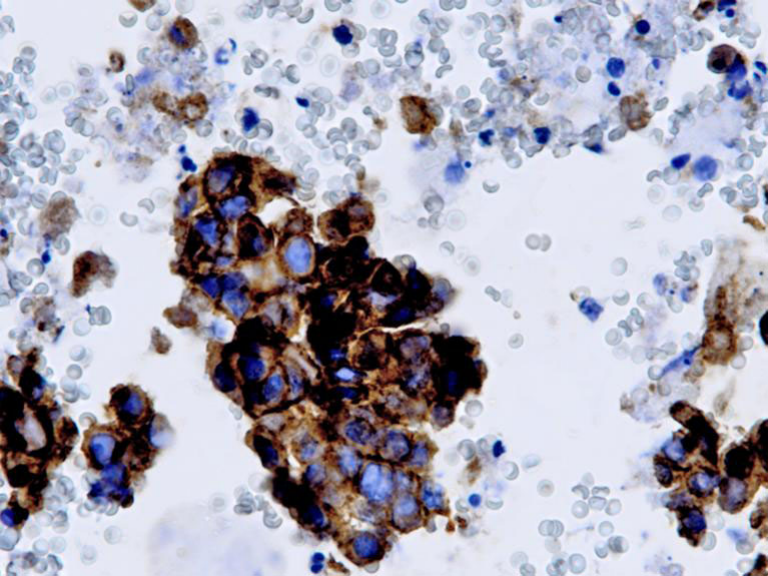
**Immunocytochemical evaluation of EGFR on non small cell lung carcinoma**. Immunohistochemistry for EGFR in large cell carcinoma (LCC) FNAC cell block evidencing a strong membrane immunoreactivity (score 3+). Original magnification ×400.

According to the IHC scoring system, 16 cases (8/20 NSCLC and 8/13 pulmonary mCRC) showed an intense EGFR-immunoreactivity (score 3+) (fig 2), 5 moderate reactivity (score 2+) and 3 weak reactivity (score 1+). No immunoreactivity (score 0) was observed in 9 cases (7 NSCLC and 2 mCRC). In particular, among the 27 polysomic cases detected by CISH (12 low polysomy, 15 high polysomy), 17 (63%) scored 2+/3+ (6 NSCLC and 11 pulmonary mCRC), and 10 (37%) scored 0/1+ (8 NSCLC and 2 pulmonary mCRC). The 2 NCSLC amplified by CISH displayed a 3+ score. We did not observe any statistically significant correlation between IHC scores and CISH (p = .85).

Furthermore, a comparison between CISH and FISH was performed. FISH evidenced 4 disomic (1.6-2.0 balanced gene and chromosome 7) (16%) and 26 polysomic (84%) cases of which 7 were trisomic (2.2-3.0 balanced gene and chromosome 7) and 19 were highly polysomic (3.1-4.4 balanced gene and chromosome 7) and 3 amplified (gene-to-chromosome 7 ratio ≥ 2). Sensitivity for CISH was 60%, specificity was 89%, the positive predictive value (PPV) was 50% and the negative predictive value (NPV) was 93% (Table 2).

**Table 2:** Comparison between immunohistochemistry, CISH and FISH in 33 cell blocks from lung FNAC

IHC score	N° of cases	CISH				FISH			
		**D**	**T**	**P**	**A**	**D**	**T**	**P**	**A**

0	9	1	5	3	0	2	2	5	0
1+	3	1	0	2	0	0	0	3	0
2+	5	1	0	4	0	0	0	5	0
3+	16	2	7	6	2	2	5	6	3

Total	33	5	12	14	2	4	7	19	3

IHC: immunohistochemistry; CISH: chromogenic *in situ *hybridization; FISH: fluorescence *in situ *hybridization;

D: disomy, 1.6-2.0 balanced gene and chromosome 7; T: trisomy, 2.2-3.0 balanced gene and chromosome 7; P: polysomy, 3.1-4.4 balanced gene and chromosome 7; A: amplified, gene-to-chromosome 7 ratio ≥2

Sensitivity 60%; Specificity 89%; Positive predictive value 50%; Negative predictive value 93%

Table 3 reported the correlation between EGFR gene and chromosome 7 balanced polysomy by CISH and FISH. The overall concordance between FISH and CISH results was 97%. We observed 30 out of 33 cases not amplified (NA) and 2 NCSLC amplified (A) with both assays. CISH presented a gene-to-chromosome 7 ratio of 2.5 and 3 respectively and FISH a gene-to-chromosome 7 ratio of 2.8 and 3.3 respectively. Although there was a very low number of amplified cases, the 2 NSCLC FNAC with gene amplification by CISH were highly polysomic and this polysomy was confirmed by FISH.

**Table 3:** Comparison between CISH and FISH in 33 cell blocks from lung FNAC

CISH			FISH		
**EGFR status**	**Chromosome 7**		**EGFR status**	**Chromosome 7**	

	*Disomy*	*Polysomy*		*Disomy*	*Polysomy*
			
31 NA	6 (19%)	25 (81%)	30 NA	4 (13%)	26 (87%)
	*Disomy*	*Polysomy*		*Disomy*	*Polysomy*
			
2 A	0	2 (100%)	3 A	1 (33%)	2 (67%)

Total 33	6	27	33	5	28

CISH: chromogenic *in situ *hybridization; FISH: fluorescence *in situ *hybridization; NA: not amplified, gene-to-chromosome 7 ratio < 2; A: amplified, gene-to-chromosome 7 ratio ≥2; Disomy: 1.6-2.0 balanced gene and chromosome 7; Polysomy: 3.1-4.4 balanced gene and chromosome 7

Concordance 97%; k = 0.78; *p *< 0.0001; Sensitivity 67%; Specificity 100%; Positive predictive value 100%; Negative predictive value 97%

Only 1 NSCLC was NA by CISH (gene-to-chromosome 7 ratio 1.2) and amplified by FISH (gene-to-chromosome ratio 2.5). The k coefficient for the inter-assay agreement was 0.78 (95% CI: 45%-100% P < 0.0001). Therefore, sensitivity for CISH was 67%, specificity was 100%, PPV was 100% and NPV was 97%.

## Discussion

The present study aimed to evaluate the effectiveness of CISH to detect EGFR GCN on FFPE sections from FNAC cell blocks obtained from NSCLC and CRC pulmonary metastases.

Our findings demonstrated that: a) lung FNAC nodules provide useful material to detect the EGFR status by *in situ *hybridization, b) the CISH technique is sensitive and specific in determining EGFR GCN, c) CISH and FISH correlate between them, while there is no association between EGFR GCN and IHC overexpression.

Previous studies already demonstrated that CISH is a useful technique for the detection of EGFR and HER2 gene amplification in breast [19] and lung cancer [18] FNAC both in conventional and in monolayered smears obtained by liquid based cytology.

Herein, we showed that the CISH analysis performed on cell blocks from lung FNAC is also a valuable method for establishing the EGFR gene content in pulmonary neoplastic nodules and, as reported by other authors [18,20], there is a close association with the results provided by FISH.

To our knowledge, no previous studies have made a direct comparison between the CISH and FISH analyses in cytological specimens from lung tumors using cell block preparation. This methodological approach could be of clinical interest in the diagnosis of lung nodules since it may reduce the undetermined diagnoses distinguishing tumor histotype known to better respond to anti-EGFR targeted therapies [21,22].

Primary lung carcinomas as well as mCRC are often unresectable [23] leading to the use of FNAC procedures or bronchoscope tissue biopsy to obtain diagnostic cellular material. However, conventional cytology has not been widely used for biological analysis, primarily due to heterogeneity within samples or to the limited percentage of tumor cells usually present in the cytological smears. The method we described may be particularly useful in patients who are not candidates for surgery and may be used also on other cytological specimens as pleural effusions or bronchoalveolar lavages. In our series of 20 primary NSCLC and 13 mCRC, CISH evidenced EGFR gene amplification only in NSCLC (2/20, 10%) and an elevated incidence of high polysomy (40% NSCLC and 53% mCRC). The low number of amplified cases we found is in line with data reported in the recent literature showing that EGFR gene is rarely amplified in human cancers. In contrast, an increased EGFR GCN with balanced polysomy is more frequent occurring in approximately 25 to 40% of patients with NSCLC or CRC [24].

Discrepancy in EGFR gene amplification between CISH and FISH was found in one NSCLC case. This discordance may be likely due to the lower polysomy observed by FISH. Therefore, an agreement of 97% (k = 0.78; p < 0.0001) between CISH and FISH was detected in the total series of 33 patients without any significant differences between primary and metastatic lung nodules.

We verified that, even though the majority of samples were assessable by both the techniques, some samples were more difficult to evaluate by FISH because of high autofluorescent background due to the presence of hemosiderin or necrosis. The use of CISH allowed a simultaneous evaluation of GCN, tumor cells and detailed surrounding tissue morphology on the same slide.

Many authors demonstrated that the increase in absolute EGFR GCN detected by FISH, both in NSCLC and in mCRC [9,13], is associated with an improved response to TKI as gefitinib or to cetuximab or panitumumab respectively. Only a few studies did not confirm this predictive value [25,26]. More recently, it has been reported that in NSCLC, EGFR gene mutation is more significantly related to the response of targeted therapy to TKI [24]. In addition, some authors [18,27,28] showed, both in bioptic and cytological specimens, that a balanced increase of EGFR gene and chromosome 7 copy number is related with specific EGFR mutations. Therefore, NSCLC presenting a EGFR balanced polysomy had a high probability of response to gefinitib. Several studies have compared whether EGFR abnormalities in NSCLC, detectable by IHC, in situ hybridization or PCR, correlate with each other or represent independent variables [9,18]. Recently, a meta-analysis of nearly 5000 cases estimated that all the three assays significantly predict the response to gefitinib in NSCLC patients [29].

Concerning mCRC, Sartore-Bianchi et al [30] suggested that EGFR disomic tumors or with low polysomy have a reduced likelihood of response to panitumumab and Moroni et al [10] proposed that the response to anti-EGFR treatment with cetuximab is strictly related to EGFR copy number. More recently, it has been reported that k-ras mutations represent the strongest predictor for cetuximab failure in EGFR-positive/FISH-negative cases [12,13]. In contrast, Campanella et al [31] showed that in mCRC patients treated with chemotherapy plus cetuximab, increased EGFR GCN was significantly associated with a better clinical outcome, independent of k-ras status.

The lack of correlation between GCN and EGFR overexpression both in NSCLC and mCRC confirms current opinion that EGFR IHC positivity does not allow to accurately select patients eligible for anti-EGFR treatment [24].

In conclusion, our findings indicate that CISH is a valid method to detect EGFR GCN, also on cell blocks of lung FNAC and could be used as an alternative to gene mutation analysis both in NSCLC and in mCRC.

## List of Abbreviation

EGFR: epidermal growth factor receptor; NSCLC: non small cell lung cancer; MoAbs: monoclonal antibodies; CRC: colorectal cancer; CISH: Chromogenic *in situ *hybridization; FISH: fluorescence *in situ *hybridization; GCN: gene copy number; FNAC: fine-needle aspiration cytology; PPV: positive predictive value; NPV: negative predictive value; TK: tyrosine kinase; TKI: tyrosine kinase inhibitors; IHC: immunohistochemistry; mCRC: metastatic colorectal cancer; SCC: squamous cell carcinomas; LCC: large cell carcinomas; ADC: adenocarcinomas; CT: computer tomography; RT: room temperature; DAB: 3'3-diaminobenzidine; FFPE: formalin fixed paraffin embedded; CEP7: chromosome enumeration probe 7; DAPI: 4',6-diamidino-2 phenylindole; NA: not amplified; A: Amplified; CI: confidence interval.

## Competing interests

The authors declare that they have no competing interests.

## Authors' contributions

GS, AM* and MM were responsible for the study design and they produced, acquired, analysed and interpreted the data and drafted the manuscript, AM was responsible for providing assistance in data acquirement and manuscript drafting, VR and MS were responsible for immunohistochemistry assay, ADB and FN were responsible for the CISH and FISH analysis and interpretation, IT was responsible for the database set up and for the statistical analyses. All authors read and approved the final manuscript.
